# Tuning of the Brillouin scattering properties in microstructured optical fibers by liquid infiltration

**DOI:** 10.1038/s41598-023-37345-0

**Published:** 2023-06-28

**Authors:** Ester Catalano, Raffaele Vallifuoco, Luigi Zeni, Alexis Dufour, Emmanuel Marin, Sylvain Girard, Aldo Minardo

**Affiliations:** 1 Department of Engineering, Università della Campania Luigi Vanvitelli, Via Roma 29, 81031 Aversa, Italy; 2 Laboratoire Hubert Curien, UMR CNRS 5516, 18 Rue du Professeur Benoît Lauras, 42000 Saint-Etienne, France

**Keywords:** Engineering, Electrical and electronic engineering

## Abstract

We demonstrate the possibility to modify the Brillouin scattering properties of a microstructured pure-silica core optical fiber, by infiltrating a liquid inside its holes. In particular, we show that the dependence of the Brillouin frequency shift (BFS) on the temperature can be reduced by infiltration, owing to the large negative thermo-optic coefficient of the liquid. Infiltrating a chloroform-acetonitrile mixture with a refractive index of 1.365 inside the holes of a suspended-core fiber with a core diameter of 3 µm, the BFS temperature sensing coefficient is reduced by ≈ 21%, while the strain sensitivity remains almost unaltered. Besides tuning the temperature sensing coefficient, the proposed platform could find other applications in Brillouin sensing, such as distributed electrical and magnetic measurements, or enhanced Brillouin gain in fibers infiltrated with high nonlinear optical media.

## Introduction

Distributed optical fiber temperature and strain sensors based on stimulated Brillouin scattering (SBS) have been widely used in several application fields, including structural health monitoring and security asset integrity, in virtue of their long sensing range and high spatial resolution^1–6^. Despite these advantages, conventional SBS-based sensors suffer from the temperature and strain cross-sensitivity of the Brillouin frequency shift (BFS). Several methods have been proposed to solve this crucial issue, broadly falling into two categories: the methods aiming at discriminating the strain and temperature effects by measuring both, and those which aim at reducing or even canceling out the sensitivity to one of the two parameters. The first category includes the use of fibers with multiple acoustic^7–9^ and/or optical modes^10^. While relatively simple to implement, these methods usually exhibit poor discrimination capabilities, with condition numbers of the sensing coefficient matrix in the order of a few hundreds. A more effective technique relies on the simultaneous measurement of the BFS and birefringence in polarization-maintaining fibers^11^. However, this method requires the use of a complex setup with precise alignment of the optical beams injected into the sensing fiber. Other methods rely on the measurement of the Brillouin scattering at two widely separated wavelengths^12^, or on the combined use of multiple scattering phenomena such as Brillouin and Rayleigh scattering^13,14^, or Brillouin and Raman scattering^15^. The second category includes athermal Brillouin sensing in specialty fibers such as sapphire-derived^16^, lithium aluminosilicate^17^, or highly Ge-doped silica fibers^18^. While effective, these fibers usually exhibit a low Brillouin gain and/or large attenuation, which limits their applicability to only a few meters. In particular, the sapphire-derived^16^ and lithium aluminosilicate^17^ optical fibers have a low Brillouin gain and a high attenuation loss (> 200 dB/km). Instead, the highly doped Ge-doped fiber reported in Ref.^18^ (fiber HNL-98) has a high Brillouin gain (about seven times larger than SMF-28), while the attenuation loss is still large (200 dB/km).

Strain-insensitive Brillouin temperature sensing has been demonstrated as well, based on gas-filled hollow core fibers^19^. For the latter, the zero-strain sensitivity is paired with a large Brillouin gain, which turns out to be 6 times higher than in a standard SMF.

In this paper, we utilize a microstructured optical fiber whose Brillouin scattering properties are manipulated by liquid infiltration. A suspended-core fiber (SCF) with a 3-µm pure silica core is used for experiments. The design of this fiber ensures a high overlap of the guided mode with the air holes running along its length, making it particularly efficient as a sensing platform^20–22^. We show, both numerically and experimentally, that filling the holes of a SCF with a liquid having a specific refractive index, the temperature sensing coefficient can be reduced, while keeping the strain sensing coefficient almost unchanged. By filling the holes with a chloroform-acetonitrile mixture with a refractive index of 1.365, the thermal sensitivity of the BFS is reduced from 931 kHz/°C to 733 kHz/°C, while the strain coefficient remains almost unaltered (≈39 kHz/µε).

## Principle of operation

Due to SBS, two lightwaves counterpropagating along a single-mode optical fiber (the pump wave and the Stokes wave) induce an intense acoustic wave through electrostriction, if their frequency shift is close to a value, known as BFS, given by^23^:

1 $$BFS=\frac{2{n}_{eff}{V}_{a}}{\lambda }$$

where ${n}_{eff}$ is the effective refractive index of the optical mode, ${V}_{a}$ is the acoustic velocity of the medium, and $\lambda$ is the optical wavelength of the pump in vacuum. When condition (1) is met, the electrostrictively-driven acoustic wave backscatters the pump wave, reinforcing the Stokes wave. Therefore, the process manifests itself as a moving-Bragg-type coupling between the pump and the Stokes wave. In standard single-mode fibers (SMF), the BFS is influenced by both strain and temperature, mostly due to the induced changes in the acoustic velocity^24,25^. For not excessively wide strain and temperature variations, the BFS change is a linear combination of these two effects:

2 $$BFS={BFS}_{ref}+{C}_{T}\Delta T+{C}_{\varepsilon }\Delta \varepsilon $$

where ${BFS}_{ref}$ is the BFS at a reference condition (typically, at room temperature and zero strain), ${C}_{T}$ (${C}_{\varepsilon }$) is the temperature (strain) sensing coefficient, $\Delta T$ ($\Delta \varepsilon$) is the temperature (strain) change from the reference condition. At the pump wavelength of 1.55 µm, the BFS in a standard silica SMF is ≈ 10.85 GHz, the temperature coefficient ${C}_{T}$ is ≈ 1 MHz/°C, and the strain coefficient ${C}_{\varepsilon }$ is ≈ 50 kHz/µε^24,25^. Equation (2) puts in evidence the challenge of temperature/strain discrimination in conventional optical fibers.

In a microstructured optical fiber, the infiltration of a gas or a liquid inside its holes may induce a change in its Brillouin properties. Recently, a gas-filled hollow core fiber has been shown to exhibit a highly enhanced Brillouin gain, allowing strain-free high-performance distributed temperature sensing^19^. The Brillouin scattering can be even excited in the gas using the evanescent field in a nanofiber^26^. In these cases, the Brillouin scattering occurs in the gas medium. In this paper, instead, we rely on the Brillouin scattering excited into the solid core of a suspended-core fiber. Backward Brillouin scattering has been already observed in solid-core microstructured optical fibers^6,27,28^; however, the modification of the Brillouin properties by liquid infiltration has not been demonstrated yet. In our experiments, the presence of a liquid into the holey structure of the SCF has the only function of modifying the effective refractive index of the optical mode, through overlap of its evanescent tail with the liquid. Furthermore, the temperature and strain sensing coefficients are modified as well. Specifically, we can express the temperature sensing coefficient as^29^:

3 $${C}_{T}=\frac{dBFS}{dT}=\frac{2{n}_{eff}}{\lambda }\frac{d{V}_{a}}{dT}+\frac{2{V}_{a}}{\lambda }\frac{d{n}_{eff}}{dT}=\frac{2{n}_{eff}}{\lambda }TAC+\frac{2{V}_{a}}{\lambda }TOC={C}_{T1}+{C}_{T2}$$

where $TAC=\frac{d{V}_{a}}{dT}$ is the thermo-acoustic coefficient, and $TOC=\frac{d{n}_{eff}}{dT}$ is the thermo-optic coefficient. In a standard SMF, the TAC value is 0.555 m s^−1^ K^−1^, while the TOC is 10.4 × 10^–6^ K^−1^^29^. Using the acoustic velocity in silica ${V}_{a}$ = 5970 m s^−1^, and an effective refractive index of 1.45, the temperature coefficients ${C}_{T1}$ and ${C}_{T2}$ become 1038 and 80 kHz °C^−1^, respectively at 1.55 µm wavelength. Apparently, the change in the acoustic properties dominates the dependence of the BFS from temperature. An equation similar to Eq. (3) holds true for the strain sensitivity, which is decomposed in a term related to the strain-acoustic coefficient (SAC), and another one related to the strain-optic coefficient (SOC):

4 $${C}_{\varepsilon }=\frac{dBFS}{d\varepsilon }=\frac{2{n}_{eff}}{\lambda }\frac{d{V}_{a}}{d\varepsilon }+\frac{2{V}_{a}}{\lambda }\frac{d{n}_{eff}}{d\varepsilon }=\frac{2{n}_{eff}}{\lambda }SAC+\frac{2{V}_{a}}{\lambda }SOC={C}_{\varepsilon 1}+{C}_{\varepsilon 2}$$

By using SAC = 29.2 km s^−1^ ε^−1^ and SOC = − 0.262 ε^−1^^29^, the strain coefficients ${C}_{\varepsilon 1}$ and ${C}_{\varepsilon 2}$ for a silica SMF are calculated as 55 and − 2 kHz με^−1^, respectively at 1.55 µm wavelength.

**Figure 1:**
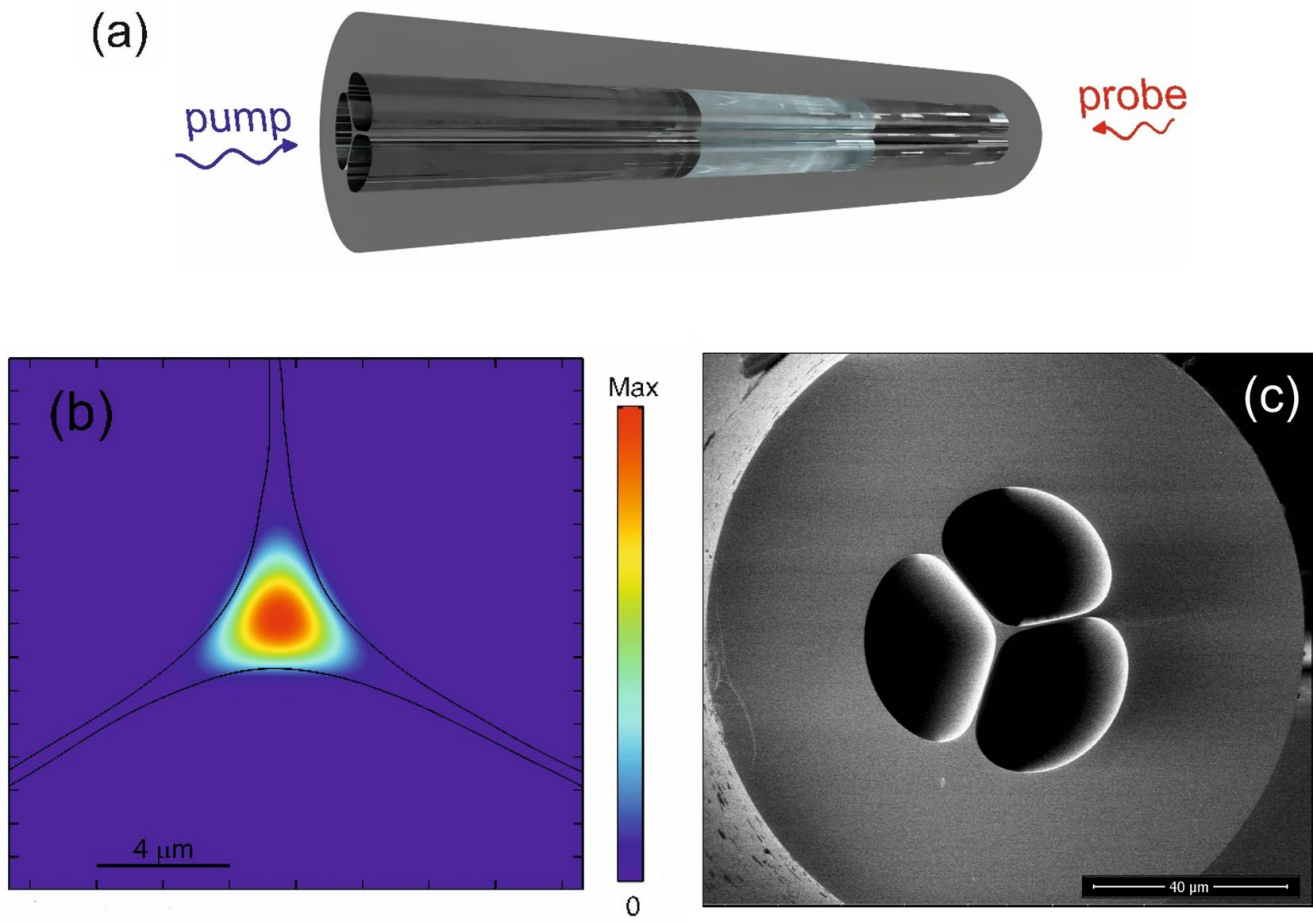
(**a**) Suspended-core fiber used for experiments; (**b**) electrical field distribution across the non-infiltrated SCF at λ = 1550 nm; (**c**) SEM transversal view of the SCF.

In a liquid-filled suspended-core fiber such as the one illustrated in Fig. 1a, the TAC can be assumed as the same of a silica SMF, as the acoustic wave is excited into the solid core. Instead, the TOC is influenced by the infiltrated liquid, by an amount depending on the thermo-optic coefficient of the liquid, as well as the fraction of evanescent field filling the holes. Therefore, by properly choosing the infiltrated liquid, the TOC can be adjusted and, with it, the temperature coefficient ${C}_{T2}$. Note that, the liquid infiltration also modifies the coefficient ${C}_{T1}$, due to the change in ${n}_{eff}$.

**Figure 2:**
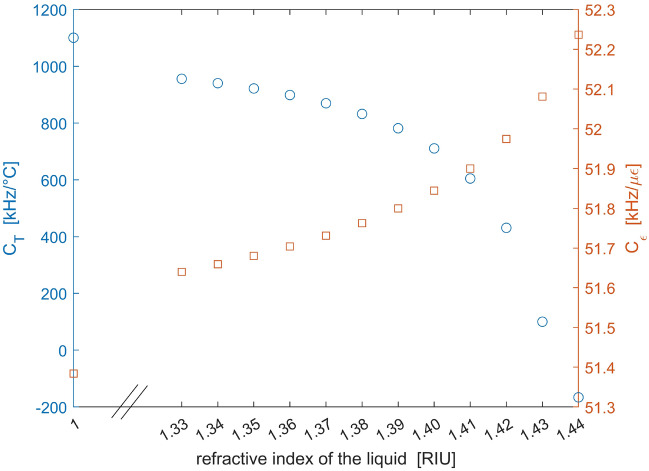
Temperature and strain sensing coefficients in the SCF, computed using Eqs. (3)–(4) and FEM simulations.

Finite-element-method (FEM) simulations were performed to obtain the effective optical refractive index of the SCF as a function of the temperature and refractive index of the infiltrated liquid (see Fig. 1b as an example of FEM-computed profile of the SCF optical mode at 1550 nm). Our SCF presents a pure silica core with a diameter of 3 μm (± 0.3 μm), and three large air holes with a diameter of ≈ 26 μm (see Fig. 1c). We report in Fig. 2 the temperature and strain sensing coefficients calculated by inserting in Eqs. (3–4) the FEM-derived TOC and ${n}_{eff}$ values, as a function of the refractive index of the infiltrated liquid from 1.33 to 1.44. The results for non-infiltrated holes (n = 1) are included as well. A thermo-optic coefficient of $-4 \cdot {10}^{-4}$°C^−1^ has been assumed for the infiltrated liquid^30^. We must remark that the non-infiltrated SCF presents a birefringence of (according to FEM simulations) B ≈ 1.8 × 10^–4^, which progressively decreases with the refractive index of the filling liquid. For example, B ≈ 5.7 × 10^–5^ for n = 1.3348. In the following, we neglect the SCF modal birefringence, as it has a negligible impact on its Brillouin frequency shift. The results shown in Fig. 2 indicate that, varying the refractive index from n = 1 (air) to n = 1.44, the strain sensitivity slightly increases, because of the increase in ${n}_{eff}$ (see Eq. (4)). This variation, however, is only ≈ 1.7% over the whole investigated range. Instead, the temperature sensing coefficient changes sensibly. Remarkably, the BFS thermal sensitivity approaches zero by infiltrating a liquid with a refractive index of ≈ 1.43. In such a case, up to ≈ 35% of the light field intensity propagates into the fiber holes at λ = 1550 nm. The numerical results therefore indicate the feasibility of athermal Brillouin strain sensing using a liquid-infiltrated microstructured fiber.

## Results and discussion

**Figure 3:**
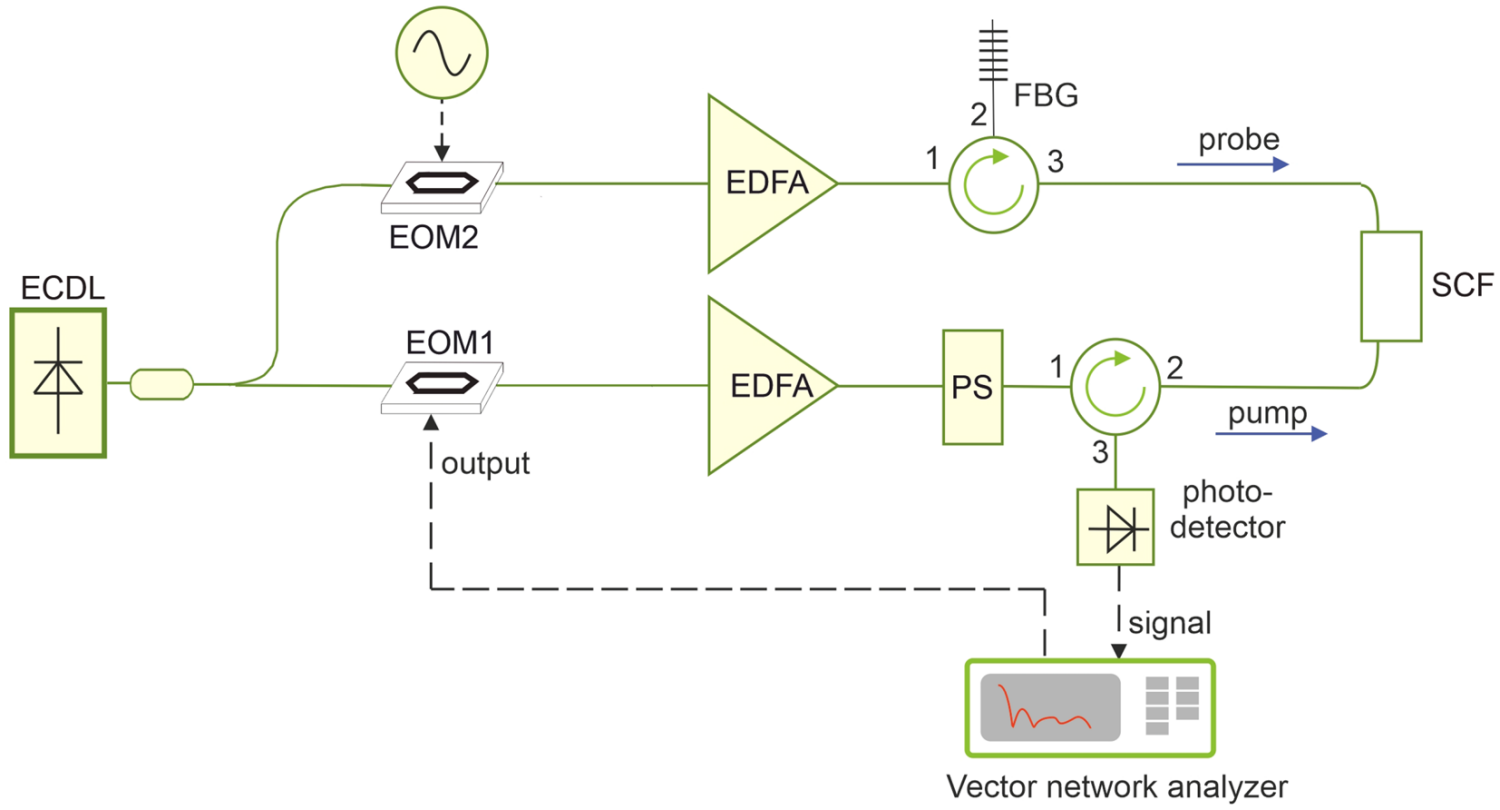
Schematic of the experimental setup for Brillouin optical frequency-domain analysis measurements. (*ECDL* external cavity diode laser, *EOM* electro-optic modulator, *EDFA* Erbium doped fiber amplifier, *FBG* Fiber Bragg Grating, *PS* polarization switch, *SCF* suspended-core fiber).

A high spatial resolution Brillouin Optical Frequency-Domain Analysis (BOFDA) setup^3,31^ was used to acquire the BFS distribution along a 3-m piece of SCF (Photonics Bretagne). The implemented scheme is depicted in Fig. 3. The output of an external-cavity diode laser (ECDL) at 1.55 µm is divided in two branches. The beam in the lower branch is modulated by an electro-optic modulator (EOM1) biased at its quadrature point and driven by the output of a vector network analyzer (VNA). Then, the modulated beam is amplified with an Er-doped fiber amplifier (EDFA) to 19 dBm and injected into the SCF as the pump light. The beam in the upper branch is modulated by another electro-optic modulator (EOM2), biased at its null point, and driven by a multi-GHz microwave generator to generate two sidebands. The upper sideband is filtered out using a fiber Bragg grating (FBG), while the lower sideband is amplified by another EDFA and launched into the other end of the SCF, acting as the probe light. When the microwave frequency falls within the Brillouin gain spectrum of the sensing fiber, the transmitted probe light acquires an intensity modulation which is recorded by the VNA in amplitude and phase. By sweeping the VNA frequency, spatially resolved Brillouin scattering measurements are obtained with a two-point spatial resolution dictated by the maximum modulation frequency^31^. Since our VNA operates up to 20 GHz, a spatial resolution as low as 5 mm can be obtained. The polarization dependence of the Brillouin gain is compensated using a polarization switch (PS), which rotates alternatively the state-of-polarization of the pump light by π/2. The baseband transfer function is then acquired and averaged over two orthogonal polarizations of the pump light. Finally, the Brillouin gain spatial distribution is obtained by applying an inverse Fourier transform to the acquired data and converting the time coordinate into a spatial one using $z=\frac{{v}_{g}}{2}t$, ${v}_{g}$ being the group velocity of the optical guided mode.

**Figure 4:**
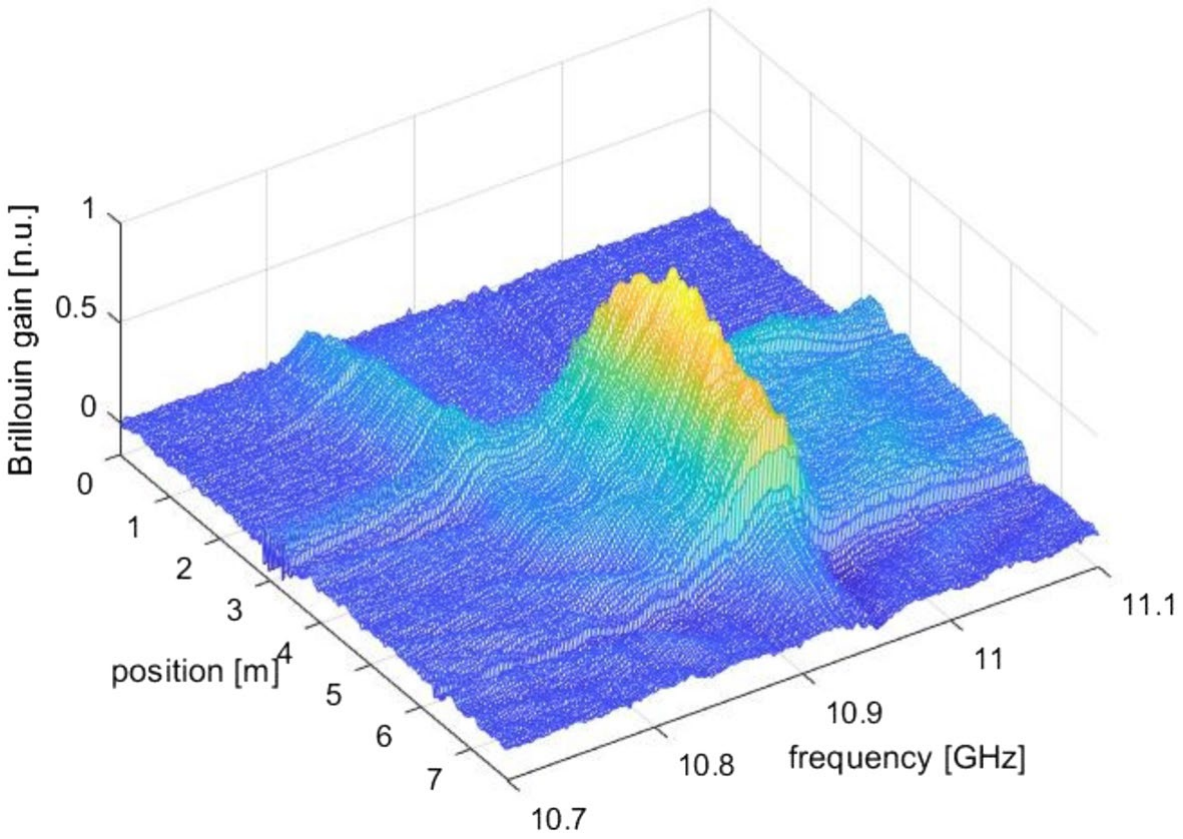
Brillouin gain distribution along the 3 m long non-infiltrated SCF. The first 3.25 m correspond to the SMF circulator pigtail and the lensed fiber.

Due to large difference between the mode-field diameter (MFD) of the circulators’ SMF pigtails (≈ 10.5 µm) and the MFD of our SCF (≈ 3 µm), a pair of lensed fibers, manufactured by OZ Optics and with a spot diameter of 2.5 µm, were employed to launch the pump and probe lights into the SCF through butt-coupling. After optimal alignment, the estimated coupling loss was ≈ 2 dB per side. Figure 4 shows the Brillouin gain distribution acquired at a spatial resolution of 6.20 cm (i.e., by scanning the VNA frequency up to ≈ 1.6 GHz). In the recorded traces, the first 3.25 m correspond to the Brillouin scattering along the SMF circulator pigtail and the lensed fiber, while the subsequent 3 m correspond to the SCF sample. The averaged BFS along the SCF was ≈ 10.927 GHz, in a reasonable agreement with the Brillouin frequency shift estimated by using ${V}_{a }=5970$ m s^−1^ and the FEM-calculated ${n}_{eff}=1.4173$ (BFS = 10.918 GHz). It is also observed that the Brillouin peak gain in the SCF was ≈ 6 dB higher than in the standard SMF, in virtue of its smaller acousto-optic effective area^32^.

**Figure 5:**
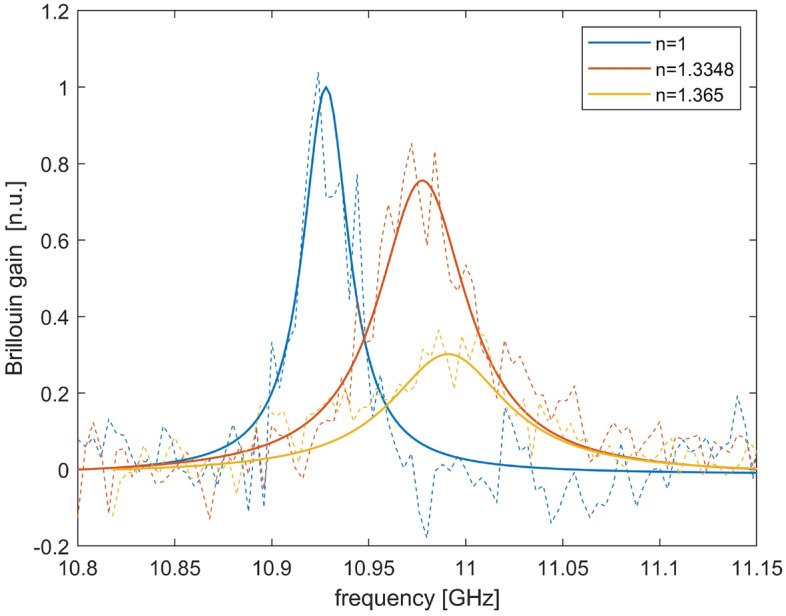
Brillouin gain spectra acquired for different infiltrations of the SCF, and corresponding Lorentzian fit curves.

A piece of the SCF was then infiltrated, by simply dipping one of the two ends into a liquid for ≈ 1 min, while leaving the other end open at room pressure. Owing to the capillarity effect, about 20 cm of the SCF was infiltrated. The Brillouin gain spectra were then acquired over the entire SCF at a spatial resolution of 3.10 cm. Two liquids were chosen: acetonitrile, with a refractive index of 1.3348 at 1.55 µm wavelength^33^, and an acetonitrile-chloroform mixture with a refractive index of 1.365 (as determined by an Abbe refractometer). These two solvents were chosen owing to their low absorption at 1.55 µm (9.81 × 10^–3^^34^ and 20.9 × 10^–3^ cm^−1^^35^ for acetonitrile and chloroform, respectively). After acetonitrile infiltration, the fiber was put in oven at T = 85 °C for 2 h to evacuate the holes and proceed with a new infiltration. The measured SBS gain spectra of the non-infiltrated and infiltrated SCF are plotted in Fig. 5 (experimental data and Lorentzian fitting curves). The blue shift resulting from liquid infiltration is clearly seen: the BFS increased by 49 MHz when passing from n = 1 (air) to n = 1.3348, or 63 MHz when passing from n = 1 to n = 1.365. These values are in reasonable agreement with those obtained using Eq. (1) and the FEM-calculated effective refractive indexes ($\Delta BFS$ = 54 and 66 MHz, respectively). The Brillouin linewidth is also shown to broaden upon liquid infiltration: the full-width at half-maximum (FWHM) obtained by Lorentzian fitting is 31 MHz for the non-infiltrated fiber, 56 MHz for the fiber infiltrated with acetonitrile (n = 1.3348), and 74 MHz for the fiber infiltrated with the acetonitrile-chloroform mixture (n = 1.365). The progressive broadening of the Brillouin gain spectrum is thought to originate from the roughness-induced acoustic attenuation at the silica/holes interfaces, which has a larger impact on the spectrum linewidth when the evanescent field is enhanced by liquid infiltration^36,37^. The low SNR exhibited by the spectra shown in Fig. 5 may be attributed, on one side to the high spatial resolution used for our tests (3.10 cm), on the other side to the coupling and propagation loss of our suspended core fiber. As regards the first point, the use of a longer fiber could allow the measurements to be performed at a coarser spatial resolution, with a proportionally beneficial effect in terms of SNR. As regards the second point, a different fiber should be adopted, characterized by a reduced scattering loss. Finally, we mention that some optimization of the measurement setup in Fig. 3 could be beneficial as well. For example, the probe light could be realized using a single-side band modulator (SSB) driven with an amplified microwave signal, thus avoiding the use of an EDFA on the probe branch and its related ASE noise. Similarly, the EOM in the pump branch (EOM1) could be driven by an RF driver boosting the power of the frequency swept signal produced by the VNA. Please note that, for our tests the VNA output at 6 dBm was directly used to drive EOM1, which resulted in a modulation depth as small as ≈ 0.3 rad. The amplification of the VNA output would increase the modulation depth of the pump, and consequently the modulation signal impressed to the probe by SBS.

**Figure 6:**
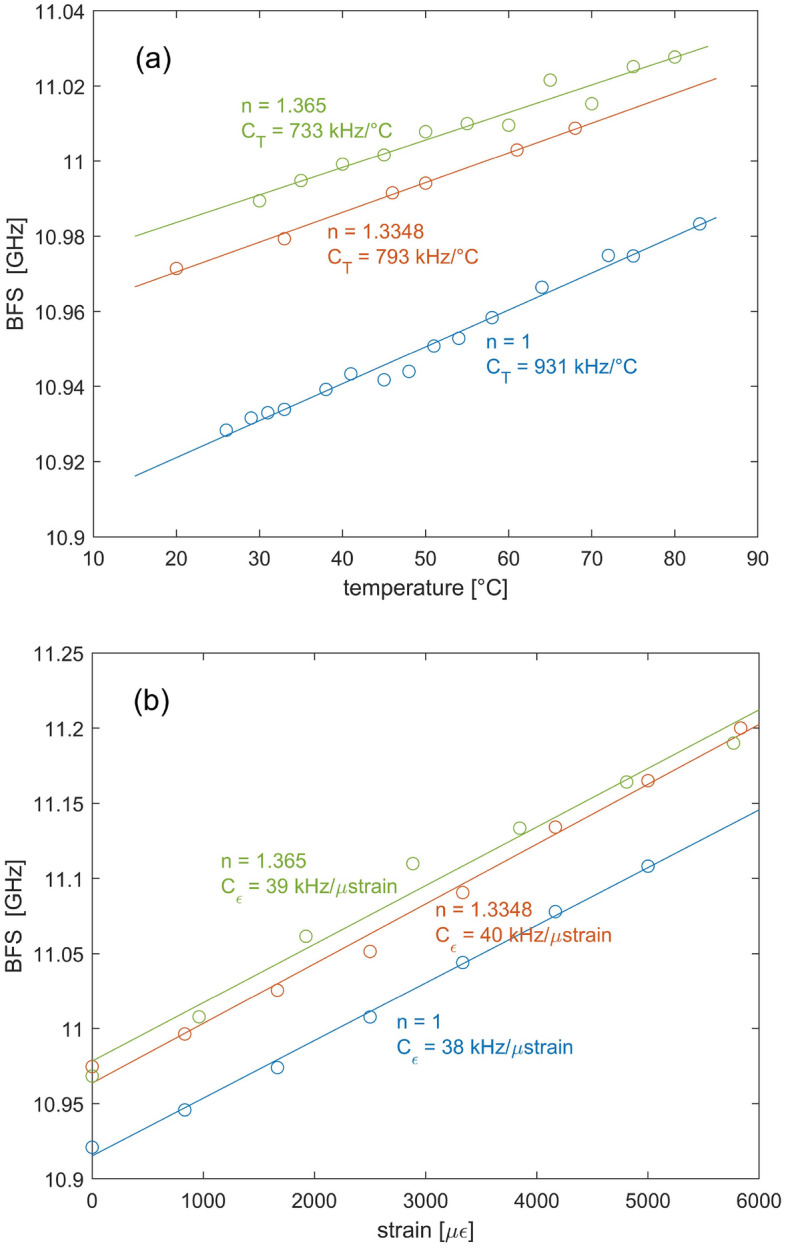
Brillouin frequency shift dependence on (**a**) temperature and (**b**) strain, for the non-infiltrated and infiltrated SCF.

The BFS dependences on strain and temperature for the non-infiltrated or infiltrated SCF were then analyzed. To this aim, a piece of fiber was either heated by immersion in hot water or strained by attaching its ends to two micro-positioners. The Brillouin gain spectra at the heated or strained positions were acquired with a spatial resolution of 3.10 cm, while their peak gain frequency was estimated using a quadratic fitting. We show in Fig. 6 the measured BFS at the heated (or strained) position, together with the results of linear fitting. The figures reveal that, the strain sensitivity is almost unchanged upon liquid infiltration, being 38 ± 2 kHz/με for the non-infiltrated fiber, 40 ± 3 kHz/με for the fiber infiltrated with acetonitrile (n = 1.3348), and 39 ± 6 kHz/με for the fiber infiltrated with the acetonitrile-chloroform mixture (n = 1.365). Instead, the temperature sensitivity is progressively reduced as the refractive index of the infiltrated medium increases. In fact, a higher refractive index of the filling liquid implies that a largest portion of the optical mode overlaps with it, whose negative thermo-optic coefficient partially counteracts the BFS increase deriving from the increase in the acoustic velocity [see Eq. (3)]. The linear fits indicate a temperature coefficient of 931 ± 70 kHz/°C for the non-infiltrated fiber, 793 ± 64 kHz/°C for the fiber infiltrated with acetonitrile (n = 1.3348), and 733 ± 121 kHz/°C for the fiber infiltrated with the acetonitrile-chloroform mixture (n = 1.365). Comparing these values with those computed using Eq. (3) and shown in Fig. 2, we see that the experimental coefficients are lower, probably due to some discrepancy between the actual values of the material constants (acoustic velocity, TAC and TOC) and those assumed for the calculations. Nonetheless, the relative variation of the temperature sensing coefficients is consistent with the numerical predictions: experimentally, the temperature coefficient variation between the non-infiltrated and the two infiltrated fibers is 138 and 198 kHz/°C, respectively, while the corresponding numerical values are 150 and 201 kHz/°C.

We have also tried to infiltrate acetonitrile-chloroform mixtures with a higher refractive index, aiming at reducing further the temperature sensitivity. However, in those cases the resulting optical loss was too large to get reliable BFS measurements. The increase of optical loss with the refractive index of the infiltrated medium is attributed to the higher roughness-induced scattering optical loss resulting from the stronger evanescent field. As discussed in Ref.^38^, the roughness-induced loss is the main cause of attenuation in our SCF. Therefore, a fiber with a reduced sensitivity to scattering loss shall be used in a future demonstration, in order to obtain the double objective of reducing the loss while increasing the refractive index of the infiltrated medium (therefore further reducing the BFS thermal sensitivity).

## Conclusions

We have demonstrated, both numerically and experimentally, the possibility to tune, by liquid infiltration, the Brillouin temperature sensing coefficient in a microstructured optical fiber. While the largest observed variation of the temperature coefficient was only ≈ 21%, the achieved results show promise in the use of infiltrated microstructured fibers for athermal Brillouin strain sensing. Furthermore, although the adopted fiber exhibits a high attenuation loss (≈ 200 dB/km at 1.55 µm, according to the manufacturer), microstructured holey fibers with a loss of a few dB/km^39,40^ are available. This would make the proposed approach suitable for sensing lengths up to a few hundred meters, although, for such lengths, a proper infiltration method should be devised. For example, the substance could be infiltrated as a gas at high pressure along the fiber heated at a high temperature, while condensing at the (lower) operating temperature. The proposed approach paves the way for many potential sensing applications, such as distributed electrical field sensing by liquid crystal infiltration^41^, magnetic field sensing using magnetic fluid infiltration^20,42^, or enhanced Brillouin sensing using highly nonlinear liquid infiltration^43^. The SBS could be even excited into the liquid through the evanescent field of the guided mode^26^: due to the much lower acoustic velocity in the liquid, the BFS of the liquid would be well separated by the BFS of silica. This would provide a mechanism for simultaneous strain and temperature measurements based on the different sensitivities of the Brillouin resonances into the liquid and into the solid core.

## Data Availability

The data that support the findings of this study are available from the corresponding author upon reasonable request.
